# Supplementary figures and images for: Attenuation parameter and liver stiffness measurement using FibroTouch vs Fibroscan in patients with chronic liver disease
Source: PLoS One. 2021 May 3;16(5):e0250300. doi: 10.1371/journal.pone.0250300 (PMC8092664; doi:10.1371/journal.pone.0250300)

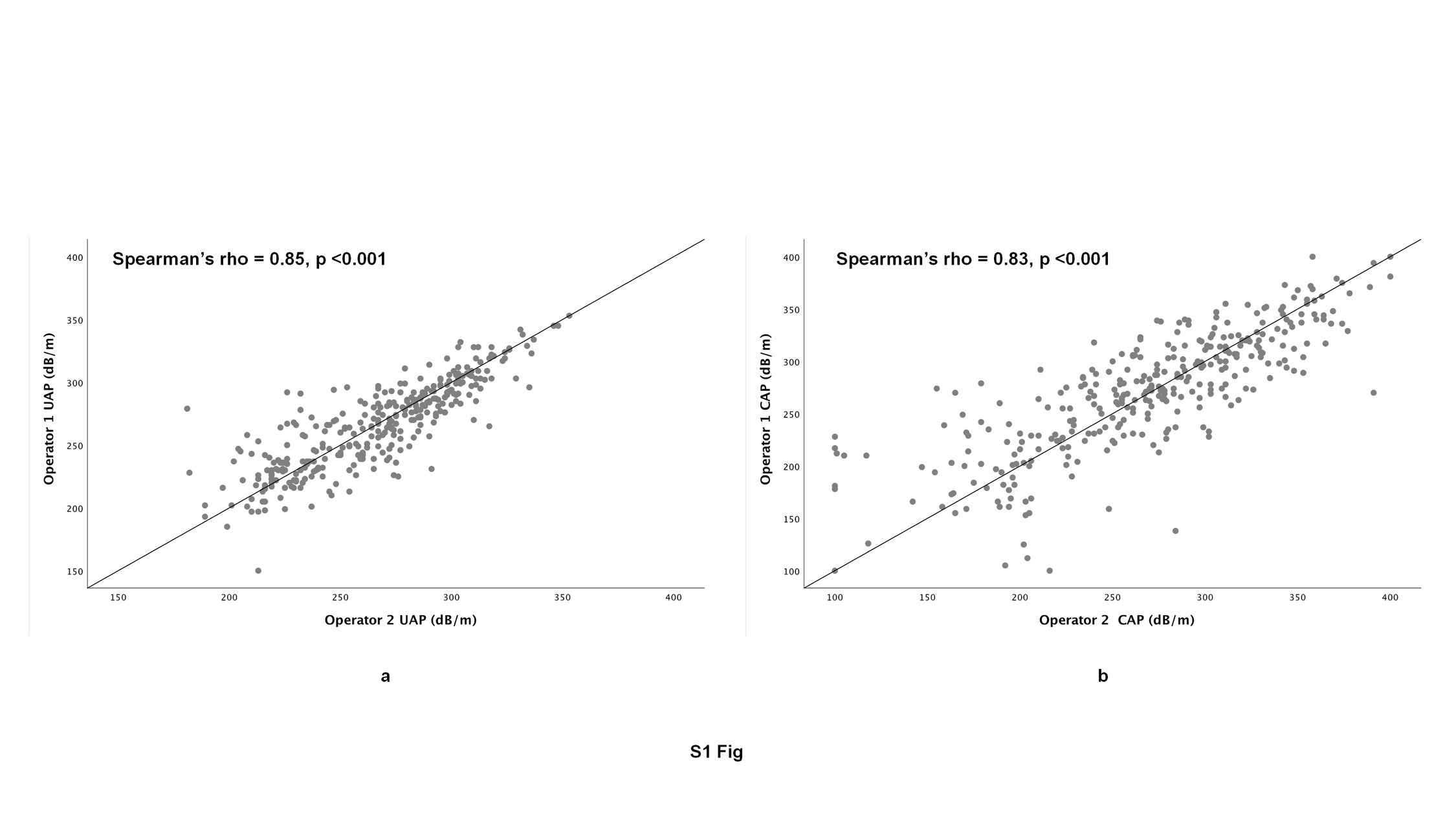

Supplement: S1 Fig — Scatter plots of attenuation parameters obtained by operator 1 vs operator 2 using (a) Fibrotouch, and (b) Fibroscan. (TIF) [file pone.0250300.s001.tif]

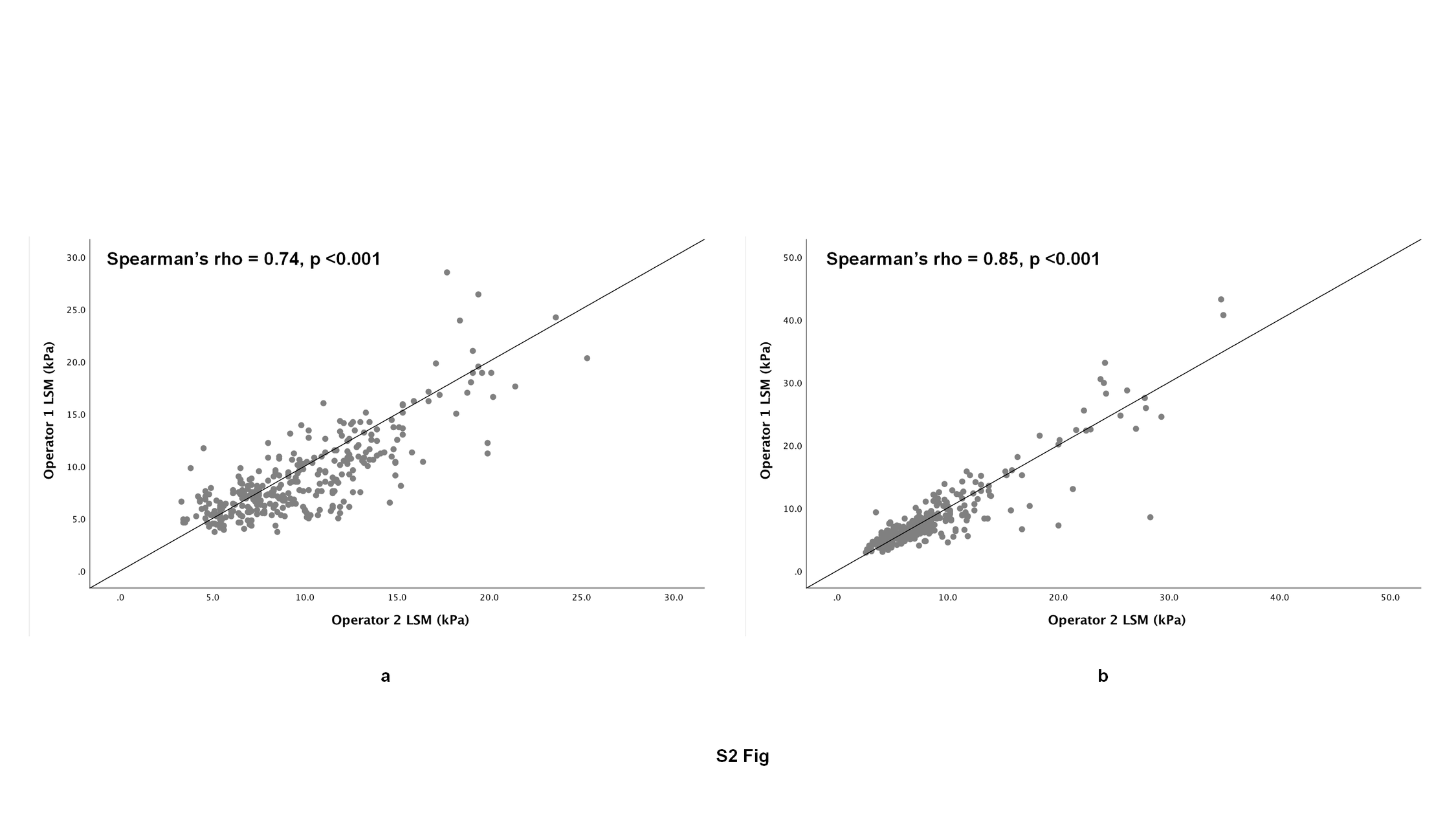

Supplement: S2 Fig — Scatter plots of liver stiffness measurements obtained by operator 1 vs operator 2 using (a) Fibrotouch, and (b) Fibroscan. (TIF) [file pone.0250300.s002.tif]
