# Supplementary material for: Attenuation parameter and liver stiffness measurement using FibroTouch vs Fibroscan in patients with chronic liver disease
Source: PLoS One. 2021 May 3;16(5):e0250300. doi: 10.1371/journal.pone.0250300 (PMC8092664; doi:10.1371/journal.pone.0250300)
Supplement: S1 Table — (DOCX) [file pone.0250300.s003.docx]

**S1 Table** Summary of studies that evaluated the performance of Fibroscan (FS) and FibroTouch (FT)

| **Authors, Year** | **Study Population** | **Findings** |
| --- | --- | --- |
| Yuan et al, 2014 | 75 patients with chronic hepatitis B underwent FS and FT examinations.  46 of them were selected for liver biopsy.  Patients with BMI >30 kg per m^2^ were excluded.  Mean BMI of study population 21.4 kg per m^2^ | Pearson’s correlation for LSM obtained using FT and FS was 0.99 (p <0.05).  Based on liver biopsy, the AUROC of FT for diagnosis of fibrosis stage ≥F1, ≥F2, ≥F3, and ≥F4 was 0.89, 0.94, 0.901 and 0.91, respectively. |
| Ou et al, 2015 | 211 patients with chronic hepatitis B underwent FS and two FT examinations. One of the FT examination was performed using the same site as FS.  Patients with BMI >28 kg per m^2^ were excluded | Pearson’s correlations for LSM obtained using FT and FS were >0.8 (p <0.05). |
| Zhang et al, 2016 | 66 patients with primary biliary cirrhosis underwent FS and FT examinations and liver biopsy. | Spearman’s correlation between FT or FS and fibrosis stage determined by liver biopsy were 0.90 and 0.88, respectively (both p <0.01).  There were no significant differences in the diagnostic performance between FT and FS in determining fibrosis stage. |
| Zeng et al, 2016 | 1621 patients with chronic liver disease of various aetiologies and also healthy subjects underwent FS and FT examinations.  Mean BMI of study population 23.8 kg per m^2^ | Pearson’s correlation for LSM obtained using FT and FS was 0.645 (p <0.01).  Pearson’s correlation for attenuation parameter obtained using FT and FS was 0.620 (p <0.01). |
| Chen et al, 2017 | 313 patients with chronic hepatitis B underwent FS and FT examinations and liver biopsy. | Pearson’s correlation for LSM obtained using FT and FS was 0.858 (p <0.01)  Based on liver biopsy, the AUROC of FT for diagnosis of fibrosis stage ≥F3, ≥F4, ≥F5, and ≥F6 was 0.915, 0.856, 0.839 and 0.816, respectively, while the AUROC of FS for diagnosis of fibrosis stage ≥F3, ≥F4, ≥F5, and ≥F6 was 0.933, 0.883, 0.849 and 0.856, respectively. |
| Xu et al, 2019 | 435 patients with chronic liver disease of various aetiologies underwent FS and FT examinations and liver biopsy.  Majority of patients had chronic hepatitis B (54.5%). Only a small number of patients had NAFLD (3.4%)  Mean BMI of study population 22.9 kg per m^2^ | Pearson’s correlation for LSM obtained using FT and FS was 0.85 (p<0.001)  Based on liver biopsy, the AUROC of FT for diagnosis of fibrosis stage ≥F2, ≥F3, and ≥F4 was 0.84, 0.92, and 0.93, respectively, while the AUROC of FS for diagnosis of fibrosis stage ≥F2, ≥F3, and ≥F4 was 0.86, 0.91, and 0.96, respectively. |
