# Supplementary material for: Attenuation parameter and liver stiffness measurement using FibroTouch vs Fibroscan in patients with chronic liver disease
Source: PLoS One. 2021 May 3;16(5):e0250300. doi: 10.1371/journal.pone.0250300 (PMC8092664; doi:10.1371/journal.pone.0250300)
Supplement: Attachment — Submitted filename: Response to Reviewer Comments.docx [file pone.0250300.s005.docx]

18^th^ March 2021

Professor Daisuke Tokuhara

Academic Editor

PLOS ONE

**Ms. Ref. No.: PONE-D-20-33839**

**Title: Attenuation parameter and liver stiffness measurement using FibroTouch vs Fibroscan in patients with chronic liver disease**

We would like to thank the editor and reviewers for the invaluable comments that have helped to improve the quality of our manuscript. Following are the point-to-point responses to the comments:

Reviewer #1: The authors have performed a meticulous study to compare results of FS with FT across a spectrum of etiologies and disease severities. However some issues remain:

In abstract it is mentioned that there were 0 invalid measurements with FT vs 2 with FS, but in results it is mentioned that there were 2 failed with FS alone, and 1 with FS and FT both, meaning 3 invalid with FS and 1 with FT. Please clarify.

Reply: We regret that these details were not clear in the originally submitted manuscript. The FS examination was unsuccessful (i.e., <10 valid measurements were obtained after 30 attempts) all four times (examination was performed two times by each of the two operators) in three patients. The FT examination was unsuccessful (i.e., <10 valid measurements were obtained after 30 attempts) all four times (examination was performed two times by each of the two operators) in one patient (who also had unsuccessful FS all four times). Comparison cannot be made without any successful FS and/or FT examination. Therefore, these three patients were excluded from the analysis. On the other hand, the 0 invalid measurements for FT and median 2 (IQR 1 - 4) invalid measurements for FS (presented in Table 3) are the average numbers of invalid measurements for the examinations performed on the 163 patients included in the analysis. The three patients who did not have any successful Fibroscan and/or FibroTouch examinations were excluded from the analysis. We have included a note on this below Table 3 for clarity. In addition, we have revised the abstract for clarity by stating the number of patients and examinations that were included in the analysis instead of the number of patients who were enrolled in the study and the number of attempted examinations. We have also explicitly state that 163 patients with 1304 examinations were included in the analysis in the results section of the revised manuscript.

While correlation between FS and FT is good, the absolute values in a given patient are quite significantly different both for attenuation parameter and LSM specially for cases in the lowest and highest ranges. Hence cut-offs for FS and FT cannot be same. FS has well validated cut-off, but FT cut-offs need to be validated with liver biopsies.

Reply: Thank you for this comment. We completely agree and have already mentioned similar points in the discussion section of the originally submitted manuscript, albeit in separate paragraphs.

*“These findings suggest that FT and FS results may not be interchangeable, although they are strongly correlated and may be similarly used for evaluation of patients with chronic liver disease.”*

*“Nevertheless, further studies on FT using liver biopsy as reference standard may be needed in view of our findings of only moderate agreement in FT and FS diagnosis when applying previously established cut-offs for FS.”*

The authors have used LSM cut-offs of <10, >15 and >20 in this study for absence of cirrhosis, presence of cirrhosis and presence of significant portal HTN. But this difference is usually apparent after USG and endoscopy. In real life we often use further refined cut-offs between 6 and 15KPa to separate out various stages of fibrosis from F0 to F4 since it is necessary to determine whether a given patient has significant or advanced fibrosis or not without resorting to liver biopsy. Comparison of FS and FT using these cut-offs would be clinically more relevant. As per my experience FT tends to have higher LSM values in this range.

Reply: Thank you for this comment. Previous studies on different populations have found different liver stiffness measurement cut-offs for the diagnosis of different fibrosis stages. Importantly, there is substantial overlap in liver stiffness measurement between adjacent fibrosis stages, and the selection of cut-offs is based on trade-off between sensitivity and specificity. To simplify matters, the Baveno VI Consensus Workshop has recommended liver stiffness measurement <10 kPa to rule-out compensated advanced chronic liver disease, 10-15 kPa to be suggestive of compensated advanced chronic liver disease, >15 kPa to be highly suggestive of compensated advanced chronic liver disease, and ≥20-25 kPa to be having clinically significant portal hypertension (PMID: 26047908). We have also validated the 10 kPa and 15 kPa cut-offs in our study on NAFLD patients (PMID: 30658997). Therefore, we have used these cut-offs in our current study. However, in response to this comment, we have included subgroup analysis for patients with chronic hepatitis B infection and for patients with NAFLD. The number of patients with chronic liver disease of other aetiologies is too small for subgroup analysis. We have used the 8 kPa and 11 kPa cut-offs for significant fibrosis and cirrhosis, respectively, for patients with chronic hepatitis B infection (PMID: 26563120), and the 7.0 kPa and 10.3 kPa cut-offs for significant fibrosis and cirrhosis, respectively, for patients with NAFLD (PMID: 20101745). We have included the results in the revised manuscript, updated the methods section and cited the references accordingly.

Taking validated FS cut-offs as the gold standard (since it is better validated than FT), the sensitivity, specificity and accuracy of FT for predicting various stages of fibrosis as well as for predicting portal hypertension should be calculated to make this study more clinically meaningful.

Reply: Thank you for this comment. We have included the AUROC, optimal cut-off, sensitivity, specificity, positive predictive value, negative predictive value, and accuracy of FT using validated FS cut-offs as the gold standard in the revised manuscript as suggested and updated the methods section accordingly.

Reviewer #2: The current study strictly compare the parameters between FibroScan and FibroTouch. I have no comments on the data and the data-based conclusions. But there is a limitation should be described adn discussed. FibroScan provided S, M and XL probe, thus S probe can be applied to children. In addition, children has age-dependent different reference value in LSM that was disclosed by the accumulated studies (Liver International. 2020;40:2602-2611; PLoS One. 2016;11:e0166683). On the other hand, FibroTouch has only one type of probe. I think FibroTouch's probe will not be applied to the narrow intercostal space of the children. Or, a future study is required for the FibroTouch to evaluate the applicapability in children. Authors are required to include the discussion or limitation of the study regarding the above point (applicapability in children) by citing the recent above references.

Reply: Thank you for this comment. We have included these points in the discussion section of the revised manuscript and cited the references accordingly.

*“Finally, the results of this study may not be applicable to the paediatric population, especially when children have been shown to have age-dependent increases in liver stiffness measurement. Further studies are needed to investigate the use of FibroTouch in the paediatric population.”*

In addition, we have written non-alcoholic fatty liver disease in full when it appeared in the manuscript for the first time. We have also made some edits to the structure of several sentences in the abstract so that it is less similar to the abstract of this work that was submitted for poster presentation at The Liver Meeting Digital Experience 2020 and subsequently published in the supplementary issue of Hepatology. We have obtained approval from Wiley to publish the amended abstract along with this full text of our work, which has not been published elsewhere before.

Once again, we would like to thank the editor and reviewers for the comments put forth to help us improve on our manuscript. We have tried our very best to answer to the comments and to make all the necessary changes to the manuscript and hope that it will be acceptable to the editors and reviewers.

Thank you very much and looking forward to hearing from you!

Best regards,

Dr Chan Wah Kheong

Professor and Senior Consultant Gastroenterologist and Hepatologist

Department of Medicine, Faculty of Medicine, University of Malaya, 50603 Kuala Lumpur, Malaysia

Telephone no.: +60379492965

Fax no.: +60379604190

E-mail: [wahkheong2003@hotmail.com](mailto:wahkheong2003@hotmail.com)
